# Supplementary material for: Off-Gassing of Semi-Volatile Organic Compounds from Fire-Fighters’ Uniforms in Private Vehicles—A Pilot Study
Source: Int J Environ Res Public Health. 2021 Mar 16;18(6):3030. doi: 10.3390/ijerph18063030 (PMC8001179; doi:10.3390/ijerph18063030)
Supplement: Supplementary file 1 [file ijerph-18-03030-s001.pdf]

Table S1. Concentrations of PAHs, OPFRs and PBDEs in structural firefighting uniforms (ng g^{-1}).

	Laboratory Tests			Vehicle	MDL
	40 °C	60 °C	80 °C		
Phe	130	170	160	100	6.6
Ant	14	32	29	15	0.11
Flu	48	58	73	61	2.4
Pyr	66	79	99	62	2.9
BaA+Chr	33	38	62	46	0.92
BbF+BkF	27	33	53	30	0.17
BeP	11	16	25	13	0.50
BaP	13	17	19	10	0.55
I123cdP	8.3	10	19	14	0.24
DahA	1.7	1.5	3.2	3.1	0.13
BghiP	9.9	13	24	15	0.22
$\sum_{13}$ PAHs	360	470	570	370	
TDCIPP	<MDL	<MDL	<MDL	<MDL	28
TBOEP	0.2	3.9	<MDL	17	0.077
TPhP	1000	1900	1200	370	0.69
EHDPP	240	880	130	910	46
TEHP	0.27	0.62	0.58	13	0.047
TMPP	4.4	2.7	10	34	0.16
$\sum_{6}$ OPFRs	1200	2800	1300	1400	
BDE-28	<MDL	<MDL	<MDL	<MDL	0.67
BDE-47	<MDL	<MDL	<MDL	210	4.6
BDE-100	<MDL	<MDL	0.92	47	0.67
BDE-99	<MDL	<MDL	<MDL	280	8.1
BDE-154	<MDL	<MDL	<MDL	6.5	4.6
BDE-153	0.73	<MDL	<MDL	8.6	0.67
BDE-183	<MDL	<MDL	<MDL	1.2	0.67
$\sum_{7}$ PBDEs	0.73		0.92	550	

Table S2. Rate of PAHs, OPFRs and PBDEs off-gassing from structural firefighting equipment (ng $uniform^{-1}$ day^{-1}).

	Laboratory Tests			Vehicle	MDL
	40 °C	60 °C	80 °C		
Phe	8300	6100	19000	8200	1100
Ant	53	1100	1900	2000	3
Flu	520	510	930	830	340
Pyr	1500	<MDL	900	970	550
BaA+Chr	27	24	25	45	7.3
BbF+BkF	16	5.90	13	9.8	1.1
BeP	4.9	4.70	5.1	5.5	0.09
BaP	6.4	4.30	3.2	3.5	0.61
I123cdP	1.7	1.80	2.0	1.40	0.14
DahA	0.21	0.20	0.59	0.76	0.05
BghiP	2.9	1.6	2.4	2.7	0.20
$\sum_{13}$ PAHs	10000	7800	23000	12000	
TDCIPP	26	<MDL	22	8.8	0.65
TBOEP	<MDL	<MDL	8.4	<MDL	5.9
TPhP	1500	580	970	1000	3.8
EHDPP	<MDL	<MDL	460	160	130
TEHP	1.3	0.38	5.8	2.1	0.029
TMPP	70	43	100	20	1.5
$\sum_{8}$ OPFRs	1600	620	1600	1200	

BDE-28	<MDL	<MDL	<MDL	<MDL	0.64
BDE-47	0.65	1.7	3.3	53	0.10
BDE-100	<MDL	<MDL	<MDL	<MDL	0.10
BDE-99	<MDL	<MDL	<MDL	<MDL	0.10
BDE-154	<MDL	<MDL	<MDL	<MDL	0.10
BDE-153	<MDL	<MDL	<MDL	<MDL	0.10
BDE-183	<MDL	<MDL	<MDL	<MDL	0.10
$\sum_7$ PBDEs	0.65	1.7	3.3	53	

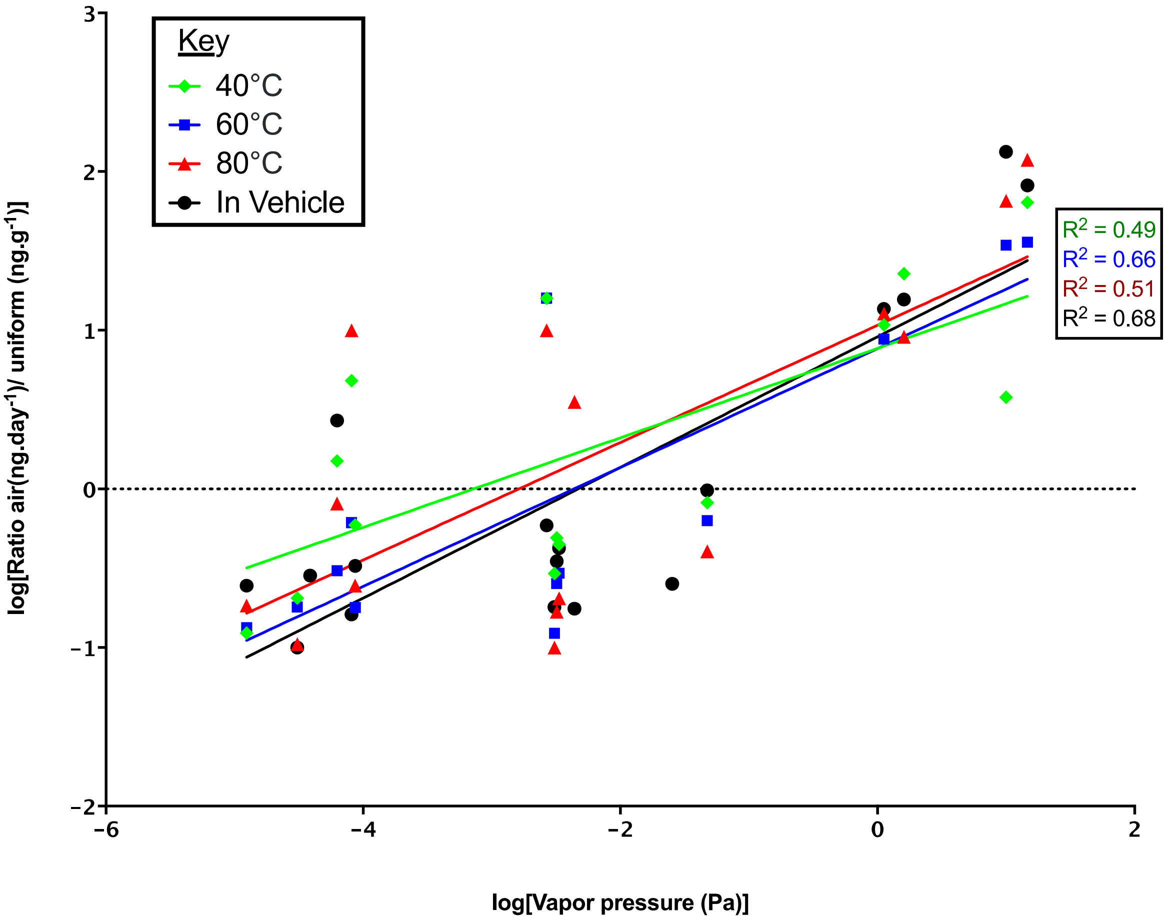

Figure S1. Relationship between vapor pressure and the rate at which SVOCs off-gassed from a structural firefighting uniform in a vehicle and in laboratory tests.
